# Reframing Nutraceuticals in Knee Osteoarthritis with Sarcopenia: A Muscle–Joint-Centered Narrative Review

**DOI:** 10.3390/nu18121871

**Published:** 2026-06-10

**Authors:** Dojoon Park, Hae-Seok Koh, Youn-Ho Choi, Jeong Wook Moon, Ilkyu Park

**Affiliations:** 1 Department of Orthopaedic Surgery, St. Vincent’s Hospital, College of Medicine, The Catholic University of Korea, Suwon 16247, Republic of Korea; onedream1106@naver.com (D.P.); vincentos@naver.com (H.-S.K.); kieek@hanmail.net (Y.-H.C.); moon5648@gmail.com (J.W.M.); 2 Department of Orthopaedic Surgery, Bucheon St. Mary’s Hospital, College of Medicine, The Catholic University of Korea, Bucheon 14647, Republic of Korea

**Keywords:** knee osteoarthritis, sarcopenia, nutraceuticals, exercise therapy, muscle strength, physical function, collagen peptides, omega-3 fatty acids, curcumin, Boswellia

## Abstract

Background/Objectives: Knee osteoarthritis (KOA) is increasingly recognized as a function-limiting condition in which pain, neuromuscular impairment, and reduced physical activity interact with sarcopenic vulnerability to accelerate functional decline. This review reappraises commonly used oral nutraceuticals through a muscle–joint framework and examines whether they can be conservatively positioned as adjuncts that reduce symptom-related barriers to exercise-based care rather than as disease-modifying therapies. Methods: This review was conducted as a structured narrative synthesis informed by SANRA principles, using a structured and transparent search process and dual-independent study selection, without quantitative meta-analysis or formal certainty-of-evidence grading. PubMed/MEDLINE, Embase, and the Cochrane Library were searched for English-language studies published from January 2000 to March 2026, supplemented by reference screening of key reviews and international guidelines. Results: Mechanistic and clinical evidence supports a plausible pathway linking KOA pain, arthrogenic muscle inhibition, reduced loading, physical inactivity, and sarcopenic vulnerability. Across glucosamine/chondroitin, collagen peptides, omega-3 fatty acids, curcumin, and Boswellia, symptomatic benefits were modest, heterogeneous, and formulation-dependent, with no consistent evidence of structural disease modification. Direct evidence that nutraceuticals improve exercise adherence or long-term physical activity remains limited; however, selected exercise-integrated or function-oriented studies show participation-relevant signals in gait speed, activity volume, and performance-based outcomes. Conclusions: Nutraceuticals should be interpreted as optional, time-limited adjuncts within exercise-centered KOA management. Their potential value lies in modest symptom support that may facilitate rehabilitation participation in selected patients, not in stand-alone treatment of KOA or sarcopenia.

## 1. Introduction

Reframing knee osteoarthritis beyond cartilage loss.

### 1.1. Knee Osteoarthritis as a Functional Disorder with Systemic Consequences

Knee osteoarthritis (KOA) is a leading cause of pain, disability, and reduced quality of life among older adults worldwide [1]. Although historically framed as a degenerative cartilage disorder, KOA is increasingly understood as a function-limiting condition in which symptoms, neuromuscular impairment, and reduced mobility are often more clinically relevant than structural pathology alone [2]. Pain, stiffness, and impaired mobility—more than imaging findings—drive patient burden, care-seeking, and healthcare utilization.

Clinically, the impact of KOA often extends beyond the joint. Persistent knee pain and functional limitation can restrict valued activities, promote sedentary behavior, and undermine participation in exercise-based care, thereby contributing to progressive functional decline [3]. These downstream consequences are particularly important in older adults, for whom sustained mobility and physical independence are central determinants of long-term health outcomes.

### 1.2. Sarcopenic Vulnerability in KOA: Definition and Clinical Relevance

Sarcopenia, characterized by age-related declines in muscle mass and strength, is a major contributor to frailty, falls, and disability in older populations [4].

In this review, the term “sarcopenic vulnerability” is used as a clinical and functional construct rather than as a diagnostic category. It does not denote established sarcopenia defined by EWGSOP2 or AWGS criteria, which require specific combinations of low muscle strength, reduced muscle quantity or quality, and/or impaired physical performance. Instead, sarcopenic vulnerability refers to an increased susceptibility to muscle weakness, reduced physical performance, and functional decline in patients with KOA, particularly in the context of KOA-related pain and reduced physical activity. This distinction is important because many patients with KOA may not meet formal diagnostic criteria for sarcopenia but may still have functionally meaningful muscle-related limitations that interfere with exercise-based care.

In patients with KOA, sarcopenia can be understood in two complementary ways. First, primary sarcopenia may coexist as an age-related condition in older patients. Second, secondary or functional sarcopenic vulnerability may be amplified by KOA-related pain, arthrogenic muscle inhibition, reduced joint loading, movement avoidance, and physical inactivity. KOA is not a primary cause of sarcopenia; however, patients with KOA frequently develop clinical conditions that increase vulnerability to muscle weakness and impaired physical performance. Pain-related inactivity, reduced joint loading, and movement avoidance can promote progressive strength loss and functional deconditioning.

Quantitatively, sarcopenia is not uncommon in KOA. Pooled estimates from KOA-only cohorts suggest a prevalence of approximately 25.1%, although substantial heterogeneity exists across diagnostic definitions and study populations [5]. In comparative analyses, KOA has been associated with approximately twofold higher odds of sarcopenia compared with age-matched controls (OR 2.07, 95% CI 1.43–3.00), underscoring a clinically meaningful overlap even in the absence of direct causality [6]. Importantly, the relevance of sarcopenia to KOA lies less in shared prevalence and more in convergent functional consequences. Lower-extremity weakness reduces the capacity to engage in exercise—the cornerstone of KOA management—thereby perpetuating a cycle of pain, inactivity, and further functional deterioration [7]. In this sense, the KOA–sarcopenia relationship is best interpreted as a bidirectional functional interaction.

Accordingly, the relationship between KOA and sarcopenic vulnerability should be interpreted at three levels of inference. First, epidemiologic studies demonstrate an association between KOA and sarcopenia or muscle-related impairment. Second, mechanistic evidence supports biological and functional plausibility through pathways involving pain, arthrogenic muscle inhibition, reduced loading, inactivity, inflammation, and anabolic resistance. Third, however, these associations and mechanisms do not establish that KOA directly causes sarcopenia, or that symptom-directed adjunctive strategies prevent it. The present review therefore uses the KOA–sarcopenic vulnerability interface as a clinically relevant functional framework rather than as a proven causal model.

Key epidemiologic and prognostic associations linking sarcopenia and KOA are summarized in Table 1.

### 1.3. Rationale for a Muscle–Joint Lens in KOA Management

Current clinical practice guidelines consistently prioritize non-pharmacological interventions, particularly structured exercise, as first-line therapy for KOA [11]. In routine care, however, implementation is often constrained by pain-related barriers, limited adherence, fear of movement, fatigue, and patient heterogeneity. This gap between guideline recommendations and real-world practice highlights the need for clinically pragmatic frameworks that integrate joint symptoms with neuromuscular function.

A muscle–joint lens addresses this need by emphasizing the reciprocal relationship between pain, muscle strength, and physical activity [12]. In this framework, KOA-related pain and inflammation may contribute to arthrogenic muscle inhibition, quadriceps weakness, reduced loading, activity restriction, and secondary functional muscle decline. These muscle-related changes may, in turn, worsen disability and reduce the patient’s ability to participate in exercise-based care. Rather than treating muscle weakness as a secondary byproduct, this perspective positions neuromuscular health as a determinant of functional outcomes and therapeutic success. It also creates a rationale for conservative adjunctive strategies that may support participation in exercise-based care—without implying direct disease modification.

Clinically, the value of this framework lies in its implications for rehabilitation rather than in redefining KOA as a primary muscle disease. Patients with KOA may fail to initiate or sustain exercise not only because of pain severity, but also because of fear of movement, low confidence in loading the affected limb, fatigue, poor balance, and reduced lower-extremity strength. These barriers are particularly relevant in older adults, in whom reduced activity may accelerate frailty-related decline and loss of independence. A muscle–joint framework therefore helps identify why symptom control alone may be insufficient unless it enables participation in strengthening, aerobic, neuromuscular, balance, and mobility-oriented exercise.

### 1.4. Scope, Novelty, and Objectives of This Narrative Review

Within this context, nutraceuticals have attracted interest as potential adjuncts in the management of KOA [13]. However, the available evidence remains inconsistent, and international clinical practice guidelines adopt cautious or conditional positions. Accordingly, the purpose of this narrative review is not to promote nutraceuticals as core treatments, disease-modifying therapies, or substitutes for exercise, but to synthesize the existing evidence through a clinically grounded, function-oriented lens that prioritizes exercise participation and patient safety.

Previous reviews of nutraceuticals in osteoarthritis have primarily focused on molecular mechanisms of action, individual supplement efficacy, or the plausibility of disease-modifying effects. While these contributions are valuable, they have generally evaluated nutraceuticals in isolation from the exercise-based management strategies that constitute first-line care. To our knowledge, no review has specifically examined nutraceuticals through the lens of exercise-participation facilitation in patients with KOA and sarcopenic vulnerability—a perspective that shifts the evaluative emphasis from direct symptomatic or structural endpoints toward functional engagement with guideline-endorsed rehabilitation.

The added value of this review is therefore not the claim that nutraceuticals exert large or disease-modifying effects in KOA. Rather, this review reframes the question around a more clinically pragmatic issue: whether selected nutraceuticals, despite modest and heterogeneous symptomatic effects, can be conservatively positioned as optional adjuncts that may reduce symptom-related barriers to exercise-centered care in selected patients. This perspective shifts the emphasis from isolated supplement efficacy toward functional engagement, rehabilitation tolerance, and preservation of mobility in patients with KOA and sarcopenic vulnerability.

Clinical question: This review examines whether selected nutraceuticals—despite limited, heterogeneous, and formulation-dependent efficacy—can be conservatively positioned as optional, time-limited adjuncts that may reduce symptom-related barriers to exercise-based care in selected patients with KOA and sarcopenic vulnerability.

To address this question, this review evaluates nutraceuticals in KOA with attention to: (1) direct effects on joint-related symptoms; (2) the plausibility and available evidence for indirect benefits mediated through physical activity, exercise tolerance, and rehabilitation participation; and (3) practical considerations relevant to real-world implementation, including safety, product variability, patient selection, and challenges in older adults.

By framing nutraceuticals as conservative, optional, and time-limited adjuncts within an exercise-centered strategy, this review aims to clarify their appropriate and limited role in contemporary KOA care, particularly for older adults with sarcopenic vulnerability.

## 2. Methods

This narrative review synthesizes current evidence on nutraceuticals relevant to knee osteoarthritis (KOA) and sarcopenic vulnerability within a muscle–joint framework, with emphasis on physical function and exercise-participation outcomes. It was designed as a structured narrative review, not as a systematic review or meta-analysis. To enhance transparency, structured search and qualitative appraisal procedures were applied; however, the synthesis remains interpretive rather than quantitative.

### 2.1. Review Design and Reporting

The structure and reporting of this review were informed by the principles of the Scale for the Assessment of Narrative Review Articles (SANRA) [14], which emphasize a clearly justified topic, explicit aims, a transparent description of the literature search, appropriate referencing, sound scientific reasoning, and balanced presentation of evidence. The review combined a structured and transparent search and selection process with a narrative qualitative synthesis, prioritizing thematic integration and clinical interpretation over quantitative pooling.

To clarify the methodological scope, the following components were performed: a structured search of multiple electronic databases; a predefined thematic framework spanning KOA symptoms, muscle and physical-function outcomes, and exercise participation; de-duplication of records; independent screening and study selection by two reviewers, with disagreements resolved through discussion and consensus; summarization of key study characteristics using common descriptive fields; and a hierarchical (tiered) approach to weighting evidence. The flow of records through identification, screening, eligibility assessment, and inclusion is documented in Supplementary Figure S1. Because this article was designed as a structured narrative review rather than a formal systematic review or meta-analysis, the following components were not undertaken: protocol registration, application of a formal per-study risk-of-bias instrument, quantitative meta-analytic pooling, and assignment of formal GRADE certainty ratings.

### 2.2. Search Strategy and Information Sources

PubMed/MEDLINE, Embase, and the Cochrane Library were searched for studies published between 1 January 2000 and 31 March 2026; the search was not updated after this date, and English-language publications were considered for inclusion. Grey literature, including conference abstracts, dissertations, preprints, and unpublished data, was not systematically searched. Searches combined controlled vocabulary (MeSH/Emtree terms where applicable) and free-text terms across three domains: (1) knee osteoarthritis; (2) nutraceuticals or dietary supplements, including collagen peptides, curcumin, Boswellia, omega-3 fatty acids, vitamin D, glucosamine, and chondroitin; and (3) sarcopenia- or muscle-related outcomes, including muscle mass, muscle strength, physical performance, gait speed, Short Physical Performance Battery (SPPB), and physical activity.

The representative PubMed query was:

(“Osteoarthritis, Knee”[MeSH Terms] OR “knee osteoarthritis”[Title/Abstract] OR gonarthrosis [Title/Abstract]) AND (nutraceutical*[Title/Abstract] OR “dietary supplement”[Title/Abstract] OR collagen [Title/Abstract] OR curcumin [Title/Abstract] OR boswellia [Title/Abstract] OR “omega-3”[Title/Abstract] OR glucosamine [Title/Abstract] OR chondroitin [Title/Abstract] OR “vitamin D”[Title/Abstract]) AND (sarcopenia [Title/Abstract] OR muscle [Title/Abstract] OR “muscle strength”[Title/Abstract] OR “physical performance”[Title/Abstract] OR “gait speed”[Title/Abstract] OR SPPB [Title/Abstract] OR “physical activity”[Title/Abstract]).

Analogous strategies were adapted for Embase and the Cochrane Library. Reference lists of included systematic reviews, major clinical guidelines, and key primary studies were additionally screened to identify further relevant publications. Records were exported to reference management software and de-duplicated before screening.

### 2.3. Eligibility Criteria (PICOS Framework)

Eligibility was organized according to a PICOS framework adapted for a narrative synthesis. Population (P): adults with KOA defined by clinical and/or radiographic criteria, including studies that additionally addressed sarcopenia, sarcopenic vulnerability, muscle weakness, or physical-function impairment. Intervention (I): oral nutraceuticals or dietary supplements relevant to KOA symptoms, muscle health, or physical function (e.g., glucosamine, chondroitin, collagen peptides, omega-3 fatty acids, curcumin, Boswellia, vitamin D, and protein- or amino-acid-based supplements). Comparator (C): placebo, active comparator, usual care, or no comparator; given the narrative scope, studies without a formal comparator were eligible when they informed mechanistic plausibility or epidemiologic associations. Outcomes (O): joint-related symptoms (pain, stiffness), patient-reported function, physical performance, muscle mass, muscle strength, exercise tolerance, or physical activity. Study design (S): randomized controlled trials, systematic reviews, meta-analyses, and major clinical guidelines were prioritized for efficacy and safety statements; prospective observational studies were included for epidemiologic or prognostic associations; and mechanistic or preclinical studies were cited only to support biological plausibility.

Studies in non-KOA older adults or sarcopenia-risk populations were considered only when they informed muscle-related biological plausibility or future research directions and were not used as direct evidence of KOA efficacy. Studies were excluded if they were non-human investigations used to support efficacy claims, narrative opinion articles without primary data, duplicate publications, or studies addressing supplements exclusively in non-musculoskeletal contexts.

### 2.4. Study Selection and Evidence Categorization

Following de-duplication, two reviewers independently screened titles and abstracts and assessed full texts against the eligibility criteria, with disagreements resolved through discussion and consensus. A total of 842 records were identified through database searching, supplemented by 56 records from reference screening. After removal of duplicates, 768 records were screened by title and abstract, of which 612 were excluded as not relevant, non-musculoskeletal, or non-nutraceutical. The remaining 156 full-text articles were assessed for eligibility, and 87 were excluded (not KOA or sarcopenia, *n* = 33; no functional or clinical outcome, *n* = 22; non-oral intervention, *n* = 14; and opinion articles or insufficient data, *n* = 18), yielding 69 sources that informed the narrative synthesis (Supplementary Figure S1). Key characteristics of the principal studies were summarized using common descriptive fields, including study design, population, intervention and formulation, comparator, relevant outcomes, effect estimates, and major limitations, to enable consistent thematic comparison across the summary tables. The synthesized evidence base comprised international clinical guidelines and consensus or position statements, systematic reviews and meta-analyses, randomized controlled trials, prospective observational studies, and mechanistic or preclinical reports used solely for biological plausibility. Because the included evidence was clinically and methodologically heterogeneous, findings were synthesized narratively rather than pooled quantitatively.

### 2.5. Evidence Appraisal and Synthesis

Evidence was organized using a hierarchical tier framework to guide the interpretive weight assigned to individual sources: Tier 1, international clinical guidelines or consensus statements; Tier 2, systematic reviews or meta-analyses; Tier 3, randomized controlled trials; Tier 4, prospective observational studies; and Tier 5, mechanistic or preclinical studies used only for biological plausibility. Conclusions in each nutraceutical section were drawn primarily from Tier 1–3 evidence; when multiple studies addressed similar questions, more recent, larger, and methodologically stronger evidence was preferentially emphasized. Tier 4–5 sources were used in a supportive or explanatory role and were not interpreted as sufficient evidence for clinical effectiveness.

Formal tool-based risk-of-bias ratings were not generated for each included study, given the narrative design and heterogeneity of the evidence base. However, key credibility signals were considered during interpretation. For randomized trials, these included randomization procedures, blinding, attrition, sample size, comparator choice, intervention duration, and outcome selection. For systematic reviews and meta-analyses, attention was paid to search comprehensiveness, heterogeneity, risk-of-bias assessment of included studies, and consistency of effect estimates. For clinical guidelines, development rigor, transparency of evidence linkage, and conflict-of-interest considerations were noted.

Formal certainty-of-evidence grading (e.g., the GRADE approach) was not performed. Instead, the overall strength of evidence for each nutraceutical was described qualitatively using plain-language descriptors such as “consistent evidence,” “limited evidence,” “conflicting evidence,” and “insufficient evidence.” These descriptors reflected an informal consideration of recurring credibility concerns—risk of bias, inconsistency across studies, indirectness of populations or outcomes, and imprecision—but were not derived from a formal grading instrument and should not be interpreted as GRADE certainty ratings. Throughout, outcomes were interpreted with emphasis on functional relevance, exercise participation, and clinically realistic adjunctive use in older adults with KOA and sarcopenic vulnerability; claims of disease modification, direct anti-sarcopenic efficacy, or exercise-replacing effects were avoided unless directly supported by clinical evidence.

## 3. Mechanistic Links Between Knee Osteoarthritis and Sarcopenia

From pain to inactivity: a clinically plausible pathway.

### 3.1. Pain, Arthrogenic Muscle Inhibition, and Reduced Loading

From a mechanistic perspective, the functional consequences linking KOA to sarcopenic vulnerability can be plausibly explained by pain-driven neuromuscular inhibition and reduced joint loading [7]. Pain is a defining symptom of KOA and a primary driver of functional limitation [3]. Beyond subjective discomfort, persistent knee pain induces arthrogenic muscle inhibition (AMI), particularly affecting the quadriceps, resulting in reduced voluntary activation and strength [15,16]. Importantly, this neuromuscular inhibition can occur even in the absence of overt muscle atrophy and contributes to early declines in functional capacity [2,15].

As pain persists, patients progressively reduce joint loading during daily activities [3]. This adaptive unloading may initially serve a protective role; however, sustained reductions in mechanical loading diminish neuromuscular activation and mechanical stimuli to periarticular muscles [7]. Over time, reduced loading accelerates muscle weakness, compromises joint stability, and reinforces a self-perpetuating cycle of pain, dysfunction, and activity avoidance [7,15].

### 3.2. Physical Inactivity, Low-Grade Inflammation, and Anabolic Resistance

Pain-driven reductions in physical activity extend beyond the knee joint and have systemic physiological consequences [3]. In older adults, physical inactivity is associated with low-grade chronic inflammation, impaired metabolic regulation, and reduced responsiveness of skeletal muscle to anabolic stimuli [17,18]. Together, these processes contribute to anabolic resistance, a central mechanism underlying age-related muscle loss [17,19,20].

In patients with KOA, the convergence of reduced physical activity and chronic inflammatory signaling creates a biological environment unfavorable to muscle maintenance [5,7,11]. While KOA itself is not a primary cause of sarcopenia, it functions as a clinically relevant catalyst by restricting movement and perpetuating inactivity [3]. This framework helps explain why patients with KOA may experience progressive muscle weakness and functional decline even in the absence of primary neuromuscular disease [15].

### 3.3. Why Muscle Loss Matters in KOA Management

Muscle strength and physical performance are critical determinants of clinical outcomes in KOA [15]. Reduced lower-extremity strength is associated with impaired mobility, increased fall risk, and diminished capacity to engage in exercise-based therapy—the cornerstone of KOA management [11,21]. Once clinically meaningful muscle weakness develops, functional recovery becomes increasingly challenging, particularly in older adults.

From a clinical perspective, this muscle–joint interaction reframes KOA as more than a localized cartilage disorder [7]. Instead, KOA can be understood as a condition with downstream effects on the neuromuscular system, mediated primarily through pain-related inactivity and reduced loading. These mechanisms provide biological plausibility rather than direct evidence that KOA causes sarcopenia [6]. Nevertheless, recognizing this pathway offers a mechanistic rationale for management strategies that prioritize maintenance of physical activity and muscle function, even when direct modification of joint structure is not achievable. Consistent with this functional framing, longitudinal data indicate that sarcopenia is more strongly associated with the development of symptomatic KOA than with purely radiographic disease (OR 2.29, 95% CI 1.42–3.71) [9], reinforcing the primacy of functional impairment over structural change.

## 4. Interventional Logic: Where Nutraceuticals Fit

Within an exercise-centered approach to KOA, the potential role of nutraceuticals is best understood not as direct disease-modifying therapy, but as conservative adjuncts that may reduce symptom-related barriers to functional participation.

**Figure 1:**
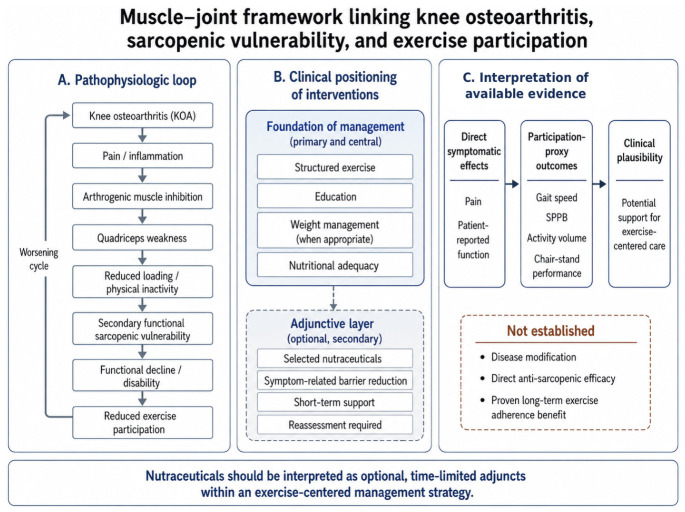
Conceptual framework linking knee osteoarthritis, sarcopenic vulnerability, and exercise participation. Panel (**A**) illustrates a clinically plausible loop in knee osteoarthritis (KOA), whereby pain and neuromuscular inhibition reduce joint loading and physical activity, contributing to muscle weakness and increased sarcopenic vulnerability. This, in turn, further limits capacity for exercise and functional recovery. Panel (**B**) positions nutraceuticals as barrier-reduction adjuncts that may modestly lower pain- or fatigue-related thresholds and support engagement in exercise-based care, without implying disease modification or independent therapeutic efficacy. Panel (**C**) summarizes the evidence ladder underpinning this framework, distinguishing between direct symptomatic effects (Table 3), observed participation-proxy outcomes such as gait speed or physical performance (Table 2), and biological or clinical plausibility. Exercise remains the central intervention throughout the framework, with nutraceuticals considered selectively and conservatively as supportive measures.

Figure 1 provides a conceptual overview of the proposed muscle–joint framework in KOA, highlighting the relationship between pain, physical inactivity, sarcopenic vulnerability, and exercise participation, and clarifying the adjunctive positioning of nutraceuticals. To operationalize this framework, participation-proxy signals observed in exercise-integrated contexts are first summarized (Table 2), followed by meta-analytic anchors that contextualize the magnitude and limitations of direct symptomatic effects (Table 3).

### 4.1. Direct Effects on Joint Symptoms

From a clinical perspective, nutraceuticals have been investigated primarily for their potential to alleviate KOA-related pain and improve patient-reported function. Current evidence indicates that certain formulations may achieve modest improvements in pain or functional scores compared with placebo [28,30]. However, effect sizes are generally small, heterogeneous across studies, and highly sensitive to product formulation, dosage, and study design. Importantly, these effects do not approach those of established pharmacological or interventional therapies when assessed across consistent outcome measures [30].

Across key nutraceuticals, meta-analytic anchors consistently fall within the small-to-moderate effect size range (Table 3), reinforcing that direct symptomatic benefits in KOA should be interpreted as modest, heterogeneous, and formulation-dependent rather than disease-modifying.

Moreover, structural modification of osteoarthritic joints has not been demonstrated in a consistent or reproducible manner [28,32]. Imaging-based outcomes, including cartilage thickness or joint space narrowing, remain largely unchanged in high-quality trials. Accordingly, nutraceuticals cannot be considered disease-modifying interventions in KOA, and any direct symptomatic benefit should be interpreted cautiously and within a limited clinical scope.

The participation-proxy outcomes summarized in Table 2 are therefore interpreted as complementary context for exercise engagement, not as direct evidence that nutraceuticals improve adherence.

### 4.2. Indirect Effects via Physical Activity and Rehabilitation Participation

Beyond direct symptom relief, nutraceuticals may exert clinically relevant effects through indirect pathways. Even modest reductions in pain can lower barriers to physical activity and rehabilitation participation in selected patients. In older adults with KOA, pain-related avoidance of movement commonly leads to reduced joint loading, progressive muscle weakness, and functional decline [23]. Interventions that facilitate sustained engagement in exercise-based care may therefore yield downstream benefits that exceed their isolated analgesic effects [22].

This indirect pathway is particularly relevant in patients at risk of sarcopenia, in whom preservation of muscle strength and physical performance is closely linked to long-term outcomes such as mobility, fall risk, and independence. Within this context, nutraceuticals may function as supportive measures that enable participation in exercise or rehabilitation programs, rather than as stand-alone treatments directed at joint pathology.

### 4.3. Why Nutraceuticals Should Be Framed as Adjuncts, Not Core Therapies

Given the modest magnitude and substantial variability of observed effects, nutraceuticals should be positioned conservatively within the broader framework of guideline-based KOA management. International clinical practice guidelines consistently emphasize non-pharmacological interventions—particularly structured exercise, weight management, and patient education—as first-line therapies [11,13]. Nutraceuticals are not endorsed as core treatments and, when mentioned, are typically classified as optional or conditionally considered due to inconsistent and heterogeneous evidence [33].

Framing nutraceuticals as adjunctive tools aligns both with the current evidence base and with real-world clinical practice. This approach avoids unrealistic expectations, mitigates the risk of overuse, and preserves the central role of exercise-based care. From an implementation perspective, nutraceuticals may be considered in carefully selected patients, particularly those in whom pain continues to limit participation in rehabilitation despite optimized conservative management.

The clinical relevance of nutraceuticals in KOA lies not in modifying joint structure, but in their potential to reduce symptom-related barriers to exercise-based care.

### 4.4. Conceptual Framework for Subsequent Evidence Synthesis

On the basis of this interventional logic, subsequent sections of this review evaluate nutraceuticals using a unified framework: (1) direct effects on KOA-related symptoms; (2) evidence relevant to muscle health or physical performance; (3) key limitations and sources of heterogeneity; and (4) clinically appropriate positioning as adjuncts within comprehensive, exercise-centered care. This structure enables transparent comparison across nutraceutical categories while maintaining a restrained and guideline-consistent interpretation. Importantly, this framework reflects a deliberate emphasis on functional outcomes and real-world clinical applicability rather than an exhaustive cataloguing of all published nutraceutical studies. The practical clinical positioning of each nutraceutical category within this framework is summarized in Table 4. Because some nutraceutical categories have limited KOA-specific evidence but potential relevance to muscle or performance outcomes, selected agents are summarized in the clinical positioning table even when they are not discussed as full standalone subsections. Vitamin D, for example, is included in the summary tables (Table 3 and Table 4) because of its general relevance to musculoskeletal health, but is not addressed as a separate evidence synthesis section because adequately powered trials have not demonstrated consistent KOA-specific symptomatic benefit in vitamin-D-replete patients, and its clinical role in KOA is primarily limited to deficiency correction rather than adjunctive symptom management within the muscle–joint framework proposed here.

## 5. Evidence Synthesis by Nutraceutical Category

The formulations, dose ranges, durations, and outcome signals reported across the cited nutraceutical studies are summarized in Table 5, underscoring the substantial heterogeneity in preparation, dose, and treatment duration that complicates cross-study interpretation.

### 5.1. Glucosamine and Chondroitin

#### 5.1.1. Effects on Knee Osteoarthritis-Related Outcomes

Glucosamine and chondroitin are among the most extensively studied nutraceuticals in the management of KOA. Multiple randomized controlled trials and meta-analyses have evaluated their effects on pain and function, yielding mixed and often controversial results. While some analyses report small but statistically significant improvements in pain and patient-reported functional outcomes compared with placebo, others demonstrate minimal or no clinically meaningful benefit, particularly when analyses are restricted to high-quality or independently funded trials [35,36].

Importantly, the direction and magnitude of benefit appear contingent on trial context and formulation. In a comprehensive Cochrane synthesis of randomized trials, chondroitin, alone or combined with glucosamine, showed small-to-moderate symptomatic benefit on pain in several comparisons, but with substantial heterogeneity and frequent concerns regarding risk of bias and sponsorship; for example, pooled estimates for pain often corresponded to roughly 8–10 points improvement on a 0–100 scale, with high inconsistency across trials [37]. Consistent with this heterogeneous signal, the NIH-sponsored GAIT trial reported no statistically significant benefit in the overall KOA cohort, while exploratory subgroup analyses suggested that combined glucosamine and chondroitin may improve pain response in participants with moderate-to-severe baseline pain [38]. More contemporary, formulation-controlled evidence further illustrates how trial design and comparator selection shape interpretation: the MOVES non-inferiority trial in symptomatic KOA with moderate-to-severe pain found that pharmaceutical-grade chondroitin sulfate plus glucosamine hydrochloride achieved pain and function improvements comparable to celecoxib over 6 months, with similar safety outcomes [34]. These findings support a cautious interpretation in which clinically meaningful symptomatic benefit—when present—may be most plausible in selected phenotypes, such as patients with higher baseline pain, and under conditions of verified product quality.

Effects vary substantially by formulation and methodological quality. Trials employing pharmaceutical-grade glucosamine sulfate have, in some instances, reported more consistent symptomatic effects than those using heterogeneous over-the-counter preparations, a distinction that has been emphasized in several European evidence syntheses [39,40]. Nevertheless, even under these conditions, reported effect sizes remain modest and clinically variable, with limited reproducibility across independent studies.

The persistent discordance between international guidelines and meta-analyses for glucosamine and chondroitin can be largely traced to three interacting factors. First, formulation heterogeneity is a major contributor: trials of pharmaceutical-grade crystalline glucosamine sulfate have reported more consistent benefit, whereas trials of glucosamine hydrochloride or heterogeneous over-the-counter preparations have more often been null; meta-analyses that pool across formulations tend to dilute any effect, whereas guidelines placing greater weight on pharmaceutical-grade preparations (e.g., ESCEO [39]) reach more favorable conclusions than those weighting the broader pooled literature (e.g., OARSI [13]). Second, trial funding and methodological quality influence results, with benefit estimates tending to be larger in smaller or industry-sponsored trials than in independently funded, rigorously blinded trials such as GAIT [38] and large network meta-analyses [36], so that pooled estimates shift depending on which trials are included. Third, differences in regulatory status—a prescription symptomatic slow-acting drug in parts of Europe versus a dietary supplement elsewhere—shape both the evidence considered and its interpretation. These factors suggest that divergent guideline positions reflect differences in evidence selection, weighting, and regulatory context more than disagreement about a large and reproducible therapeutic effect.

Importantly, available evidence does not support a consistent disease-modifying effect. Structural outcomes, including joint space narrowing and cartilage-related imaging measures, have not demonstrated reproducible improvement across well-conducted randomized trials or long-term observational analyses [13,41]. While some trials have reported statistically significant slowing of radiographic joint space loss with chondroitin in selected settings, such as small absolute differences in minimum joint space width over longer follow-up, these findings remain difficult to generalize given limited trial numbers, variability in radiographic methodology, and the broader inconsistency of structural endpoints across the literature [37]. Accordingly, glucosamine and chondroitin should not be regarded as structural modifiers of KOA, and their use cannot be justified on the basis of disease progression attenuation.

#### 5.1.2. Evidence Relevant to Muscle Health and Physical Performance

Direct evidence linking glucosamine or chondroitin to improvements in muscle mass, strength, or performance in KOA is limited. Consequently, any potential relevance to muscle health should be considered indirect and secondary, rather than a direct pharmacological effect.

In clinical contexts, modest reductions in joint pain or stiffness may facilitate greater participation in physical activity or rehabilitation programs, particularly in individuals whose activity levels are constrained by pain-related avoidance. From this perspective, glucosamine and chondroitin may be clinically relevant only insofar as modest symptom relief lowers barriers to therapeutic exercise, which remains the central intervention for preserving physical function in KOA. This concept is consistent with mechanistic “pain → inactivity → muscle weakness” pathways described in the KOA–sarcopenic vulnerability interface, in which analgesia or symptom relief may serve as a gateway to restoring loading tolerance and neuromuscular conditioning [37]. However, this pathway remains inferential and has not been directly tested in trials designed to assess muscle-specific or performance-based endpoints, such as gait speed, chair-stand performance, or composite physical performance batteries [42].

#### 5.1.3. Limitations and Sources of Heterogeneity

Interpretation of the evidence is challenged by substantial heterogeneity across studies. Variability in product composition, bioavailability, dosing strategies, duration of exposure, and regulatory oversight contributes to inconsistent findings and limits cross-trial comparability. In addition, many studies rely primarily on subjective patient-reported outcome measures, increasing susceptibility to placebo effects and expectation bias.

A further limitation lies in the discrepancy between pharmaceutical-grade formulations evaluated in controlled trials and the diverse, often poorly standardized supplements available in real-world practice. This translational gap complicates the application of trial findings to routine clinical care and partially explains divergent conclusions across meta-analyses and guideline statements. Indeed, evidence syntheses that stratify by trial characteristics frequently show attenuation of benefit estimates when restricted to larger, rigorously blinded, or independently funded trials, underscoring the sensitivity of conclusions to risk-of-bias structure [37]. Safety considerations add another layer of complexity, particularly in older adults with polypharmacy, where case reports and pharmacovigilance signals have suggested potential interactions with anticoagulant therapy, most notably warfarin [43]. Such interaction risk is a pragmatic concern in KOA populations enriched for older age and cardiovascular comorbidity, warranting explicit medication reconciliation and planned monitoring when these supplements are trialed.

#### 5.1.4. Clinical Positioning Within Comprehensive KOA Management

From a clinical perspective, glucosamine and chondroitin may be considered optional adjunctive interventions in selected patients with KOA who seek additional symptom relief despite optimized first-line conservative management. Their potential role is limited to modest symptomatic support and should not be framed as a strategy for altering disease progression or joint structure.

Consistent with international guideline positions, these agents should not replace core non-pharmacological interventions, particularly structured exercise therapy, patient education, and weight management, where appropriate. When glucosamine or chondroitin is considered, clinicians should explicitly communicate the limited and variable magnitude of benefit, the dependence on formulation quality, and the importance of predefined reassessment to avoid unnecessary long-term use. Within a comprehensive KOA management framework, any use of these nutraceuticals is best conceptualized as a time-limited, symptom-targeted trial aimed at supporting engagement with higher-value interventions rather than as a standalone therapeutic solution.

### 5.2. Collagen Peptides

#### 5.2.1. Effects on Knee Osteoarthritis-Related Outcomes

Collagen peptides have been evaluated as oral supplements intended to alleviate symptoms associated with KOA, particularly pain and functional limitation. Multiple randomized controlled trials and systematic reviews have examined their symptomatic effects, with several analyses reporting modest improvements in patient-reported pain and function compared with placebo. However, when present, these effects are generally small to moderate and appear highly sensitive to collagen source, degree of hydrolysis, dosage, and duration of supplementation, contributing to substantial between-study variability.

The clinical relevance of these findings warrants cautious interpretation. Benefits are not consistently reproduced across trials, and direct comparisons with established pharmacological treatments remain limited. In pooled randomized controlled trial evidence, collagen peptides were associated with a moderate reduction in pain versus placebo (standardized mean difference −0.58, 95% CI −0.98 to −0.18; I^2^ = 68%), although concerns regarding risk of bias, short follow-up, and between-study heterogeneity substantially limit certainty (Table 3) [28]. More recent quantitative syntheses incorporating a larger number of contemporary trials have reported statistically significant improvements in both pain and function; however, heterogeneity remains high (I^2^ often >75%), and effect sizes frequently approach—but do not uniformly exceed—established thresholds for minimal clinically important difference, underscoring the need for conservative interpretation [44].

Importantly, there is no consistent evidence that collagen peptide supplementation results in structural modification of the osteoarthritic joint. Although a small pilot randomized trial using delayed gadolinium-enhanced MRI suggested possible cartilage composition changes after collagen hydrolysate supplementation, these findings remain exploratory and have not been replicated in adequately powered trials [45]. Accordingly, collagen peptides should not be classified as disease-modifying agents in KOA.

#### 5.2.2. Evidence Relevant to Muscle Health and Physical Performance

Beyond joint-related outcomes, collagen peptides have been investigated for potential effects on muscle mass, muscle strength, and physical performance, primarily in older adults. Some studies suggest that supplementation may support gains in lean mass or muscle strength when combined with resistance exercise [46]. However, these data are derived mainly from community-dwelling or sarcopenia-risk populations without established osteoarthritis, under conditions in which exercise provides the primary anabolic stimulus.

In KOA, therefore, any muscle-related relevance of collagen peptides should be interpreted as indirect and contingent upon exercise participation. Collagen peptides may be clinically relevant only if modest symptom relief, perceived joint tolerance, or nutritional support helps patients engage in exercise-based care. There is insufficient evidence that collagen peptides independently improve gait speed, chair-stand performance, composite physical performance scores, or other sarcopenia-specific outcomes in KOA.

Importantly, the evidence base for collagen peptides—particularly for outcomes related to physical function and muscle strength—remains predominantly composed of small, heterogeneous trials, often involving industry sponsorship, product-specific funding, or commercially developed preparations. This pattern, together with short follow-up and reliance on patient-reported outcomes, warrants cautious interpretation and limits confidence that any functional or muscle-strength benefit is generalizable beyond the specific products studied.

#### 5.2.3. Limitations and Sources of Heterogeneity

The evidence base is characterized by substantial heterogeneity in formulation, dosing, study populations, and outcome measures, limiting comparability and generalizability. Preclinical evidence also supports product-level heterogeneity: in vitro studies using human osteoarthritic cartilage explants and related models have shown that collagen hydrolysate preparations may differ substantially in peptide composition and biological activity, with some preparations showing no stimulatory effect on cartilage matrix synthesis [47,48]. These findings reinforce that collagen peptide products should not be assumed to be biologically equivalent.

Many clinical trials are relatively short in duration and rely predominantly on subjective patient-reported outcome measures, increasing susceptibility to placebo and expectation effects. In addition, discrepancies between well-characterized preparations evaluated in trials and commercially available supplements complicate translation into routine clinical practice. Long-term safety data in older adults with multimorbidity or polypharmacy remain limited, further supporting a conservative interpretive approach and reinforcing the importance of predefined treatment duration and reassessment [49].

Interpretation is further limited by differences in peptide molecular weight, amino acid profile, source material, hydrolysis process, and manufacturing standards, all of which may influence bioavailability and biological effects but are inconsistently reported across trials. Sample sizes are often modest, and head-to-head comparisons between formulations are lacking. These limitations make it difficult to establish generalizable conclusions or define optimal dosing strategies for clinical use.

A further challenge lies in extrapolating findings from non-KOA populations, particularly exercise-based studies in younger or healthier individuals, to older adults with established KOA and sarcopenic vulnerability. This indirectness limits the strength of inference regarding muscle-related outcomes in the target population.

#### 5.2.4. Clinical Positioning Within Comprehensive KOA Management

In clinical practice, collagen peptides may be considered optional adjunctive interventions in selected patients with KOA, particularly those seeking additional symptom support or those with possible nutritional insufficiency. Their role should be interpreted as supportive rather than therapeutic, with expected benefits limited to modest symptom relief and possible indirect support for functional engagement.

Collagen supplementation should not be presented as a strategy for cartilage regeneration, structural modification, or independent improvement of muscle mass or strength. Instead, any potential benefit is most plausibly realized within an exercise-centered management approach, where symptom relief, perceived joint tolerance, or nutritional support may facilitate participation in rehabilitation.

Supplementation duration should be predefined, with reassessment of clinical response at a planned interval. Continued use in the absence of meaningful symptomatic or functional improvement is unlikely to provide additional benefit and may contribute to unnecessary treatment burden.

### 5.3. Omega-3 Fatty Acids

#### 5.3.1. Effects on Knee Osteoarthritis-Related Outcomes

Omega-3 fatty acids have been investigated in KOA primarily for their systemic anti-inflammatory properties, with the aim of reducing pain and improving physical function. Randomized controlled trials and systematic reviews have yielded inconsistent findings, with some studies reporting modest symptomatic benefits and others demonstrating no clinically meaningful effect. Differences in study design, background diet, comparator oils, and—critically—omega-3 formulation and dosing, including eicosapentaenoic acid (EPA) and docosahexaenoic acid (DHA) content, likely contribute to these variable results.

Where benefits are detected, effect sizes are generally small. Pooled randomized controlled trial evidence across osteoarthritis populations, including knee OA, suggests a modest improvement in pain compared with placebo (standardized mean difference −0.29, 95% CI −0.47 to −0.11; I^2^ = 60%) (Table 3), a magnitude consistent with a conservative adjunctive framing rather than a primary therapeutic strategy [29]. Functional outcomes tend to parallel pain findings, with small average improvements that are inconsistently reproduced and frequently fall below commonly accepted thresholds for minimal clinically important difference. Any observed effects are therefore unlikely to translate into reliable functional gains in routine clinical practice.

High-quality, long-term randomized evidence further tempers expectations regarding both symptomatic and structural effects. In a 24-month randomized, double-blind trial of 202 patients with symptomatic knee OA, Hill et al. found that high-dose fish oil supplementation was not superior to low-dose fish oil for WOMAC pain or function, and no between-group difference in cartilage volume loss was observed on MRI [50]. Interestingly, symptomatic improvement was greater in the low-dose comparator group, underscoring the complexity of dose–response interpretation in omega-3 supplementation. Consistent with these findings, broader evidence syntheses have not demonstrated a reproducible structural benefit of marine oil supplementation in osteoarthritis [51]. Imaging-based outcomes, including cartilage volume, cartilage thickness, or joint space measures, have not shown consistent improvement, and no convincing disease-modifying signal has emerged from available clinical trials. Accordingly, omega-3 fatty acids should not be regarded as disease-modifying interventions in KOA. Although the anti-inflammatory rationale remains biologically plausible, knee-specific clinical effects appear modest, variable, and insufficient to support disease-specific therapeutic claims.

This dissociation between mechanistic plausibility and clinical effect merits emphasis. The rationale for omega-3 fatty acids in KOA rests on systemic anti-inflammatory actions—modulation of eicosanoid and pro-resolving lipid mediator pathways and, in some contexts, reductions in circulating inflammatory markers. However, KOA pain and functional limitation are driven by a combination of local mechanical, structural, and neuromuscular factors, in which systemic low-grade inflammation is only one contributor. A systemic anti-inflammatory effect therefore does not necessarily translate into joint-specific symptomatic or structural benefit, which may explain why the biologically plausible rationale has not been matched by consistent KOA-specific clinical efficacy.

#### 5.3.2. Evidence Relevant to Muscle Health and Physical Performance

Compared with joint-specific outcomes, omega-3 fatty acids have been more extensively studied in relation to muscle health and physical performance, particularly in older adults. Experimental and clinical data suggest that omega-3 supplementation may enhance muscle protein synthesis responsiveness and support small gains in muscle strength, potentially through modulation of inflammatory signaling, membrane fluidity, and anabolic pathways when combined with resistance exercise [52,53].

However, synthesis of randomized trial evidence indicates that these effects are quantitatively small and outcome-specific. In a recent systematic review and meta-analysis including healthy young and older adults, omega-3 supplementation was associated with a very small increase in muscle strength (SMD ≈ 0.12), while no significant effects were observed for muscle mass or objective functional performance, regardless of dose (≤2 g/day vs. >2 g/day), age group, or concomitant resistance training [54]. Certainty of evidence was rated as moderate, with small-study effects and heterogeneity limiting confidence in clinical relevance.

Direct evidence linking omega-3 supplementation to improved muscle outcomes in patients with KOA remains limited. Most available data originate from non-KOA populations, often under controlled exercise conditions and in individuals without significant pain-related movement restriction. Extrapolation to KOA populations—where mobility is frequently constrained by joint symptoms and altered loading patterns—should therefore be undertaken cautiously. Consequently, the relevance of omega-3 fatty acids to sarcopenic vulnerability in KOA is best viewed as indirect and context-dependent, contingent upon the patient’s ability to engage in regular physical activity or structured exercise. Current evidence does not support omega-3 supplementation as an independent strategy for improving muscle mass, strength, or physical performance in KOA.

#### 5.3.3. Limitations and Sources of Heterogeneity

The evidence base for omega-3 fatty acids is marked by substantial heterogeneity. Variability in EPA and DHA content, dosing strategies, duration of supplementation, background dietary intake, and choice of comparator oils complicates interpretation across trials. In many studies, omega-3 formulations are insufficiently characterized, achieved plasma levels are not consistently reported, and adherence varies, limiting cross-trial comparability.

Safety considerations warrant particular attention in older adults. Although omega-3 fatty acids are generally well tolerated at moderate doses, higher-dose supplementation may be associated with gastrointestinal adverse effects and potential interactions with anticoagulant or antiplatelet therapy. Lower-dose primary-prevention trial data did not show a statistically significant increase in incident atrial fibrillation [55], whereas meta-analytic evidence from cardiovascular outcome trials has identified an atrial fibrillation signal, particularly in higher-dose supplementation settings [56]. Because these safety data derive mainly from cardiovascular rather than KOA-specific populations, they support cautious dose selection and individualized risk–benefit assessment in older adults, especially those with cardiovascular comorbidity, a history of arrhythmia, or concomitant antithrombotic therapy.

#### 5.3.4. Clinical Positioning Within Comprehensive KOA Management

From a clinical perspective, omega-3 fatty acids may be considered as adjunctive interventions in selected patients with KOA, particularly those with coexisting cardiometabolic or low-grade inflammatory comorbidities in whom omega-3 supplementation may already be under consideration for broader health indications. Within KOA care, their role is limited to modest symptomatic support and potential indirect benefits related to muscle strength when combined with exercise, rather than targeted treatment of joint pathology.

Consistent with guideline-based care, omega-3 supplementation should not replace core non-pharmacological interventions, including structured exercise therapy, patient education, and weight management. When used, decisions should be individualized, with attention to formulation, dose, comorbid conditions, concomitant medications, and realistic expectations regarding benefit. Duration of use should be bounded, with periodic reassessment to confirm ongoing relevance. From a practical standpoint, omega-3 supplementation may be most appropriately considered within a broader metabolic or cardiovascular context, rather than as a primary disease-specific intervention for KOA.

### 5.4. Curcumin and Boswellia

#### 5.4.1. Effects on Knee Osteoarthritis-Related Outcomes

Curcumin and Boswellia serrata extracts have been investigated in KOA primarily for their anti-inflammatory and analgesic properties. Multiple randomized controlled trials and systematic reviews have reported improvements in pain and patient-reported functional outcomes compared with placebo, and some analyses have suggested short-term symptomatic effects comparable to non-steroidal anti-inflammatory drugs (NSAIDs). However, these findings are inconsistent and highly dependent on study design, comparator selection, duration of follow-up, and—critically—formulation and bioavailability.

For curcumin specifically, network meta-analytic estimates have suggested improvement versus placebo on visual analogue scale (VAS) pain (mean difference −1.63, 95% CI −2.91 to −0.45) (Table 3) [30], with parallel signals for functional improvement reflected in pooled reductions in total Western Ontario and McMaster Universities Osteoarthritis Index (WOMAC) scores (mean difference −18.85 points, 95% CI −29.53 to −8.76) versus placebo (Table 3) [30]. Nevertheless, these estimates are characterized by substantial heterogeneity and potential small-study effects, as many contributing trials are short in duration, typically 4–12 weeks, and enroll relatively small samples. More recent systematic reviews and network meta-analyses that explicitly stratify by formulation have reinforced this pattern, indicating that modified or bioavailability-enhanced curcumin formulations demonstrate more consistent analgesic effects than conventional preparations, whereas unmodified curcumin often yields attenuated or non-significant benefits [57]. Thus, the apparent efficacy of curcumin should be interpreted as formulation-dependent and primarily short-term, rather than as a generalizable class effect.

Evidence for Boswellia serrata is directionally similar. Conventional and standardized boswellia extracts have shown statistically significant improvements in VAS pain, WOMAC pain, stiffness, and function compared with placebo, with pooled mean differences indicating moderate short-term symptomatic benefit, such as a WOMAC pain mean difference of approximately −14 points [31]. However, effect estimates vary widely across trials, heterogeneity is frequently high, and overall certainty is limited by methodological constraints, including risk of bias and industry sponsorship in a substantial proportion of studies [31]. These limitations support a cautious interpretation of Boswellia as a possible short-term symptom-modulating adjunct rather than a consistently reproducible analgesic intervention.

Comparisons with NSAIDs are generally indirect or based on limited head-to-head trials. Available evidence suggests that curcumin may achieve symptom relief comparable to NSAIDs over short follow-up periods, with a lower incidence of gastrointestinal adverse events, but without demonstrable superiority in efficacy and without long-term comparative data. Importantly, there is no consistent evidence that curcumin or boswellia supplementation results in structural modification of the osteoarthritic joint. Imaging-based outcomes, including cartilage thickness or joint space measures, have not demonstrated reproducible improvement across trials, indicating that these agents should not be regarded as disease-modifying therapies in KOA [58]. In contrast to short-term analgesic trials suggesting NSAID-comparable effects, long-term disease-modifying evidence remains limited.

#### 5.4.2. Evidence Relevant to Muscle Health and Physical Performance

Direct evidence linking curcumin or boswellia supplementation to improvements in muscle mass, muscle strength, or objectively measured physical performance in KOA is sparse. Although anti-inflammatory and antioxidant properties have been hypothesized to support muscle recovery or reduce exercise-associated discomfort, most human data derive from non-KOA populations, short-term experimental models, or studies focused on exercise-induced muscle damage rather than chronic mobility limitation [59].

Small randomized trials in older adults without osteoarthritis have reported modest improvements in handgrip strength, fatigue resistance, or selected performance measures with highly bioavailable curcumin formulations; however, these studies are limited by small sample sizes, short duration, and frequent industry sponsorship, and they do not address joint pain-limited mobility or altered loading patterns characteristic of KOA [60]. No randomized trials have directly evaluated curcumin or boswellia supplementation on validated physical performance endpoints—such as gait speed, chair-stand performance, or composite physical performance batteries—in KOA populations.

Accordingly, any potential contribution of curcumin or boswellia to muscle health in KOA is best interpreted as indirect, mediated through short-term symptom relief that may facilitate participation in physical activity or rehabilitation, rather than through direct anabolic, neuromuscular, or anti-sarcopenic effects. Current evidence does not support these agents as independent strategies for improving muscle mass, strength, or physical performance in KOA.

#### 5.4.3. Limitations and Sources of Heterogeneity

The evidence base for curcumin and boswellia is characterized by pronounced heterogeneity. Variability in extract composition, dosing, duration of supplementation, outcome selection, and—most importantly—bioavailability-enhancing strategies, such as phospholipid complexes, nanoparticles, or co-administration with piperine, complicates cross-trial comparison and limits reproducibility. Network meta-analytic data indicate that formulation type is a major effect modifier, with modified or enhanced formulations consistently outperforming conventional preparations in pain outcomes [57].

Methodological limitations further constrain inference. Many trials are short-term, rely heavily on subjective patient-reported outcomes, and include small samples, increasing susceptibility to placebo effects and exaggerated effect estimates. Small-study effects and publication bias have been identified in several syntheses, particularly for VAS-based pain outcomes [31,57]. Long-term safety data in older adults with multimorbidity or polypharmacy remain limited.

Safety considerations merit attention. While curcumin and boswellia are generally well tolerated at commonly studied doses, gastrointestinal adverse effects and potential interactions with anticoagulant or antiplatelet medications have been reported, particularly with higher-dose or highly bioavailable formulations [61]. Rare cases of hepatotoxicity associated with concentrated curcumin preparations have also been described, underscoring the importance of cautious product selection and monitoring.

#### 5.4.4. Clinical Positioning Within Comprehensive KOA Management

From a clinical standpoint, curcumin and boswellia may be considered adjunctive options for short-term symptom relief in selected patients with KOA, particularly those who are unable to tolerate or prefer to limit conventional analgesic therapies such as NSAIDs. Their potential role is restricted to conservative symptom management and should not be framed as modification of disease progression or joint structure.

Consistent with guideline-based care, these agents should not replace core non-pharmacological interventions, including exercise-based therapy, patient education, and activity modification. When considered, clinicians should emphasize the formulation-dependent nature of the evidence, the predominantly short-term horizon of demonstrated benefit, and the absence of disease-modifying effects. Embedding their use within a structured management plan—with predefined reassessment to avoid unnecessary prolonged supplementation—remains essential.

Overall, curcumin and Boswellia may be best positioned as short-term, symptom-targeted adjuncts in carefully selected patients, particularly when conventional analgesic use is limited or poorly tolerated. Their use should remain conservative and individualized, with attention to formulation quality, potential interactions, hepatic or gastrointestinal safety concerns, and meaningful symptomatic or functional response.

## 6. Synergistic Effects with Exercise: A Conceptual Synthesis

To clarify the strength of inference within this conceptual synthesis, the following discussion distinguishes between three levels of evidence: (1) direct effects of nutraceuticals on pain and joint-related outcomes; (2) observed improvements in physical performance or activity-related measures that may serve as proxies for participation in exercise or rehabilitation; and (3) biological and clinical plausibility supporting indirect pathways linking symptom relief to sustained physical activity. In the following synthesis, performance-based improvements are interpreted as participation-proxy or capacity-related signals, not as direct evidence that nutraceuticals improve exercise adherence or rehabilitation participation.

### 6.1. Pain Reduction and Exercise Adherence

Within this framework, exercise-based interventions remain the cornerstone of KOA management across international guidelines [11]. Nevertheless, persistent pain represents a major barrier to initiation and long-term adherence, particularly in older adults. Even modest reductions in pain may therefore carry clinical relevance if they facilitate sustained participation in physical activity or rehabilitation programs.

This logic is supported by exercise-integrated trials (Table 2): compared with resistance exercise alone, adding a protein-rich supplement was associated with an additional +0.09 m/s in walking speed over 12 weeks [22]—a magnitude approaching the ≈0.10 m/s commonly cited as a substantial, meaningful change in gait speed for older adults [27].

In the same trial, the supplemented group showed higher weekly physical activity by approximately +30.0 MET-h/week and a greater improvement in WOMAC global score (−8.21 on a 0–100 scale) (Table 2) [22], illustrating how small symptom changes may translate into functional participation.

Within this context, nutraceuticals may exert value not through their isolated analgesic effects, but by lowering symptom-related thresholds that limit exercise engagement. Across nutraceutical categories, reported improvements in pain are generally small and heterogeneous. However, when such improvements enable patients to initiate or maintain exercise-based care, the downstream functional impact may exceed what would be expected from pain reduction alone.

### 6.2. Functional Outcomes Relevant to Sarcopenic Vulnerability

The clinical importance of synergy between nutraceuticals and exercise is particularly evident in patients at risk of sarcopenia. In KOA, pain-driven inactivity contributes to progressive muscle weakness, reduced physical performance, and increased fall risk [21]. Exercise remains the primary intervention capable of addressing these outcomes, but its effectiveness is contingent on adherence and tolerability. By supporting participation in resistance or functional exercise, nutraceuticals may indirectly contribute to the preservation of muscle strength and physical performance. This indirect pathway aligns with evidence indicating that nutraceuticals alone have limited effects on muscle-related outcomes, whereas exercise remains essential. Accordingly, direct evidence linking nutraceuticals to sarcopenia-relevant functional outcomes in KOA populations remains limited.

Notably, in adults with mild KOA and concurrent sarcopenia, a 12-week nutrition intervention demonstrated a between-group improvement in physical performance of +1.03 points on the Short Physical Performance Battery (SPPB) (95% CI 0.69–1.38) (Table 2) [23], a between-group difference that approximately meets the commonly cited 1.0-point estimate for substantial meaningful change in the SPPB among older adults [27], although it reflects functional capacity rather than measured exercise adherence. This provides rare direct evidence linking nutritional support to function-relevant endpoints.

### 6.3. Why Synergy Matters More than Isolated Effect Sizes

From a clinical perspective, an exclusive focus on isolated effect sizes may underestimate the practical relevance of nutraceuticals in KOA management. Small, statistically modest improvements in pain or function may appear clinically marginal when assessed in isolation. However, when such effects enable engagement with exercise—the intervention with the strongest and most consistent evidence for improving pain, function, and physical performance—their overall contribution becomes more meaningful [62].

This reframing helps reconcile discrepancies between guideline recommendations and real-world use. Guidelines appropriately prioritize interventions with robust and reproducible benefits, while expressing caution regarding nutraceuticals because of inconsistent evidence. In practice, clinicians may encounter patients in whom symptom-related barriers preclude engagement in recommended exercise-based care. In these circumstances, nutraceuticals may serve as supportive measures that complement, rather than compete with, guideline-endorsed therapies.

### 6.4. Clinical Implications of an Exercise-Centered Synergistic Model

An exercise-centered synergistic model positions nutraceuticals as conservative adjuncts aimed at enhancing participation in core non-pharmacological interventions. This approach avoids overstatement of direct therapeutic effects, mitigates unrealistic expectations, and aligns with the realities of managing older adults with comorbidities and polypharmacy.

Importantly, this model underscores that nutraceuticals should not be considered substitutes for exercise or rehabilitation. Their use should be individualized, time-limited, and regularly reassessed in relation to functional goals and exercise adherence [33]. Framing nutraceuticals as supportive adjuncts provides a clinically coherent rationale for selective use while preserving the central role of exercise in KOA management. This synergistic model remains a hypothesis-generating framework rather than a proven causal pathway.

A critical caveat concerns whether the modest symptomatic effects of nutraceuticals are clinically meaningful enough to alter exercise behavior. Across categories, average symptomatic benefits are small, frequently below or near minimal clinically important difference thresholds, and heterogeneous. Whether such modest changes are sufficient to lower real-world barriers to initiating or sustaining exercise-based rehabilitation has not been directly demonstrated; the proposed link between symptom relief and improved participation remains a plausible hypothesis rather than an established effect. Accordingly, any anticipated adherence benefit should be interpreted cautiously, framed at the level of the individual patient, and verified through reassessment rather than assumed.

## 7. Guideline Positioning and Practice Gaps

Why recommendations diverge.

### 7.1. Overview of International Guideline Positions

Guidelines were prioritized based on international scope, methodological rigor, and relevance to non-pharmacological KOA management. Major international guidelines for KOA, including those from the American Academy of Orthopaedic Surgeons (AAOS), Osteoarthritis Research Society International (OARSI), European Alliance of Associations for Rheumatology (EULAR), and the European Society for Clinical and Economic Aspects of Osteoporosis, Osteoarthritis and Musculoskeletal Diseases (ESCEO), consistently prioritize non-pharmacological interventions as first-line therapy. Structured exercise, patient education, and weight management form the core of recommended management across all guidelines [11,33].

In contrast, nutraceuticals occupy a more variable and generally cautious position. Some guidelines do not recommend their routine use, while others allow conditional consideration in selected patients. Importantly, none endorse nutraceuticals as core or disease-modifying therapies. Notably, international guideline positions diverge in their treatment of nutraceuticals: the OARSI 2019 guideline explicitly strongly recommends against the use of glucosamine and chondroitin for KOA [13], whereas the ESCEO 2019 guideline distinguishes pharmaceutical-grade preparations and incorporates them within a stepwise pharmacologic management algorithm [39]. This variability reflects differences in evidence appraisal, outcome prioritization, and assumptions regarding product standardization rather than fundamental disagreement about their limited therapeutic role.

### 7.2. Sources of Inconsistency Across Guideline Recommendations

Several factors contribute to divergent guideline positions on nutraceuticals. First, heterogeneity in study design, formulation, and outcome measures complicates evidence synthesis. Trials often evaluate different products under the same nutraceutical label, limiting comparability and reducing confidence in pooled estimates [39]. This limitation is particularly relevant for over-the-counter supplements, where composition and bioavailability vary widely.

Second, guideline panels differ in the outcomes they prioritize. While pain and patient-reported function are commonly assessed, outcomes related to physical activity, exercise adherence, or functional participation are less consistently reported [13,39]. Consequently, potential indirect benefits—such as facilitation of exercise—are rarely captured within conventional evidence frameworks, leading to conservative recommendations that focus primarily on direct symptomatic effects.

Third, regulatory and contextual factors influence guideline interpretation. Differences in supplement regulation, quality control, and healthcare systems across regions shape assumptions regarding safety, consistency, and clinical applicability. These contextual considerations contribute to cautious or non-committal positions even when modest symptomatic benefits are observed.

### 7.3. A Conservative, Guideline-Consistent Interpretation

Taken together, current guideline positions do not reject nutraceuticals outright but emphasize uncertainty regarding the magnitude, consistency, and generalizability of benefit [33]. Interpreted conservatively, these recommendations support a framework in which nutraceuticals may be considered as optional adjuncts in selected patients, without detracting from the central role of exercise-based care. Guidance from Asia-Pacific organizations generally aligns with the global emphasis on conservative management first, with some regional nuances [63].

Importantly, this interpretation aligns with the exercise-centered synergistic model proposed in this review. By positioning nutraceuticals as supportive measures that may facilitate engagement in guideline-endorsed interventions, clinicians can remain faithful to evidence-based recommendations while addressing real-world barriers to implementation. This approach respects guideline caution, avoids overstatement of benefit, and preserves clinical flexibility in managing heterogeneous patient populations.

## 8. Safety, Quality, and Implementation Considerations

Real-world constraints in older adults.

### 8.1. Safety Signals and Dose-Related Concerns

Although nutraceuticals are often perceived as inherently safe, available evidence indicates that safety profiles vary across agents, formulations, and doses. Reported adverse effects are generally mild but become clinically relevant in older adults and at higher doses. Gastrointestinal symptoms are the most frequently reported events, while potential interactions with anticoagulant or antiplatelet therapies have been described for certain food or herbal products and should be considered in older adults with polypharmacy [64].

Importantly, safety data from randomized trials are often limited by short follow-up durations and selective inclusion criteria [30]. As a result, long-term safety in patients with multimorbidity—particularly those with chronic kidney disease, cardiovascular disease, or polypharmacy—remains insufficiently characterized. This uncertainty warrants a cautious approach comparable to that applied to pharmacological therapies, especially when nutraceuticals are considered for extended use.

### 8.2. Polypharmacy, Comorbidity, and Vulnerable Populations

Older adults with KOA frequently present with multiple comorbid conditions and concomitant medications. In such populations, the addition of nutraceuticals may increase regimen complexity and the risk of unintended interactions, particularly in patients receiving anticoagulant or antiplatelet therapy [64]. Careful consideration of dosing, duration, and patient-specific risk factors is therefore essential.

For omega-3 supplements, lower-dose primary-prevention trial data did not show a statistically significant increase in incident atrial fibrillation [55], whereas meta-analytic evidence from cardiovascular outcome trials has identified an atrial fibrillation signal, particularly in higher-dose settings [56]. Dose moderation and individualized risk review are therefore prudent in older adults with cardiovascular comorbidity, prior arrhythmia, or concomitant antithrombotic therapy.

Patients with impaired renal or hepatic function represent a vulnerable group because supplement metabolism, cumulative exposure, and interaction risks may be less predictable in routine practice. Given the limited long-term safety data in such populations, conservative use and periodic reassessment are warranted, particularly when concentrated botanical preparations or concomitant medications are involved [64,65].

### 8.3. Product Quality, Standardization, and Regulatory Variability

A major challenge in translating nutraceutical evidence into clinical practice lies in the variability of product quality and regulatory oversight. Unlike pharmaceuticals, nutraceuticals are subject to heterogeneous regulatory frameworks across regions, resulting in inconsistent standards for composition, purity, and bioavailability [39]. Products evaluated in clinical trials may differ substantially from those available commercially.

This lack of standardization complicates both efficacy and safety assessment. Clinicians cannot reliably assume equivalence across products sharing the same label, and patients may inadvertently consume formulations with uncertain dosing, bioavailability, or stability [39,66]. These issues underscore the importance of cautious interpretation of trial data and transparent communication with patients regarding uncertainties related to product quality.

### 8.4. Principles for Responsible Clinical Implementation

Given these considerations, nutraceuticals should be implemented conservatively and within a clearly defined clinical framework. Use should be individualized, time-limited, and aligned with specific functional goals, such as facilitating participation in exercise or rehabilitation [33]. Regular reassessment is essential to determine whether perceived benefits justify continued use.

Equally important is patient education. Clinicians should explicitly address the limited magnitude and variability of benefit, the absence of disease-modifying effects, and the potential for adverse events or interactions. Framing nutraceuticals as supportive adjuncts—rather than benign defaults or substitutes for evidence-based interventions—helps mitigate unrealistic expectations and reduces the risk of overtreatment.

Discontinuation should be considered if no functional or exercise-related benefit is observed within a predefined period [33].

A further implementation concern is the risk of over-medicalization. Because nutraceuticals are widely available and often perceived as benign, patients may prioritize supplementation over the interventions with the strongest evidence—structured exercise, weight management, and self-management strategies. This risk is clinically important: framed incorrectly, a symptom-directed adjunct may inadvertently displace or delay higher-value care, reduce engagement in rehabilitation, or foster the expectation that KOA can be managed pharmacologically rather than through sustained behavioral and functional change. Clinicians should therefore position nutraceuticals explicitly as optional, time-limited adjuncts that supplement—but never substitute for—exercise-based care, weight management, and patient education, and should revisit this framing at each reassessment.

In this context, “exercise-centered care” refers to specific, individualized exercise modalities rather than generic activity [11,33]. Core components include progressive resistance training to address lower-extremity weakness; low-impact aerobic activity (e.g., walking, cycling, or aquatic exercise) to support cardiovascular health and weight management; neuromuscular and balance training to improve joint stability, proprioception, and fall risk; and flexibility or range-of-motion work to preserve mobility. Programs should be individualized and progressed according to pain, baseline function, frailty, and comorbidity, with any adjunctive nutraceutical use directed at supporting participation in these components rather than at replacing them.

### 8.5. Clinical Translation: Patient Selection, Reassessment, and Discontinuation

In clinical practice, nutraceuticals may be considered only in selected patients when their intended role is clearly defined. The most appropriate candidates are patients with mild-to-moderate KOA who have persistent symptoms despite education, activity modification, and exercise-based care; patients whose pain or perceived joint intolerance limits participation in strengthening or rehabilitation; older adults with sarcopenic vulnerability or early functional decline; and patients in whom prolonged or repeated use of conventional analgesics is limited by comorbidity, tolerability, or medication-related concerns. Even in these settings, nutraceuticals should be introduced as optional adjuncts rather than as required components of KOA treatment.

A clinically responsible trial of nutraceutical use should be goal-directed, time-limited, and linked to functional outcomes. Before initiation, the expected goal should be framed in practical terms, such as improved tolerance of walking, strengthening exercise, stair negotiation, or rehabilitation participation. The aim is not to treat sarcopenia directly or modify joint structure, but to determine whether modest symptom reduction or perceived improvement in joint tolerance can support engagement with exercise-centered care. This framing is consistent with guideline-based management, in which structured exercise, patient education, weight management when appropriate, and nutritional adequacy remain the foundation of KOA care [33].

Reassessment should be planned at the time of initiation. In most patients, continued use is difficult to justify if no meaningful symptomatic or functional improvement is observed after an 8- to 12-week trial. Discontinuation should also be considered when adverse events occur, when clinically relevant drug interactions are suspected, when cost or pill burden becomes excessive, or when supplement use begins to replace exercise, rehabilitation, or appropriate medical evaluation. Progressive pain, worsening function, mechanical symptoms, or rapidly declining mobility should prompt reassessment of the underlying clinical condition rather than escalation of nutraceutical use.

Accordingly, the practical clinical role of nutraceuticals in KOA with sarcopenic vulnerability is narrow but potentially useful: they may be considered as conservative, short-term adjuncts intended to reduce symptom-related barriers to rehabilitation in selected patients. Their value should be judged by whether they help patients participate more effectively in exercise-based care, not by assumptions of disease modification, cartilage regeneration, or direct anti-sarcopenic efficacy.

## 9. Limitations

Several limitations should be considered when interpreting this review. First, although structured search and appraisal procedures were used to improve transparency, this article is a structured narrative review rather than a formal systematic review or meta-analysis. Therefore, the synthesis is interpretive rather than quantitative, and it does not provide pooled effect estimates, formal risk-of-bias ratings for each included study, or certainty-of-evidence grading. Selection bias cannot be fully excluded, particularly because the review aimed to integrate heterogeneous evidence across KOA, sarcopenia, nutraceuticals, exercise participation, and functional outcomes.

Second, the evidence base for nutraceuticals in KOA is highly heterogeneous. Studies differ substantially in supplement formulation, dose, duration, bioavailability, comparator choice, participant characteristics, and outcome selection. Products evaluated in clinical trials may also differ from commercially available preparations, limiting the direct translation of efficacy and safety findings into routine practice. These issues are particularly important for nutraceuticals because product quality and regulatory oversight vary across regions. This heterogeneity constrains direct comparison across studies and limits the strength of any pooled interpretation.

Third, most available trials focus primarily on pain and patient-reported function, whereas objective outcomes relevant to sarcopenic vulnerability and exercise participation remain underrepresented. Measures such as gait speed, chair-stand performance, Short Physical Performance Battery scores, activity monitoring, exercise tolerance, and sustained rehabilitation adherence are not consistently assessed. As a result, the proposed link between modest symptom relief and improved participation in exercise-centered care remains clinically plausible but incompletely tested.

Fourth, direct evidence in patients with KOA and confirmed sarcopenia is limited. Much of the muscle-related evidence is derived from older adults without KOA, general sarcopenia-risk populations, exercise-training studies, or mechanistic research. Extrapolation to patients with pain-limited mobility, altered loading patterns, multimorbidity, and polypharmacy should therefore be cautious. Similarly, long-term data on safety, cost-effectiveness, discontinuation strategies, and real-world adherence are insufficient.

Finally, the muscle–joint framework proposed in this review should be interpreted as hypothesis-generating rather than as proof of a causal pathway or a clinical guideline. Nutraceuticals are positioned here as optional adjuncts with a defined duration of use that may reduce symptom-related barriers to exercise participation in selected patients. This interpretation does not establish disease modification, cartilage restoration, direct anti-sarcopenic efficacy, or replacement of structured exercise and rehabilitation.

## 10. Future Directions

What evidence is actually needed?

The heterogeneity and limitations of the existing evidence on nutraceuticals in KOA partly reflect the absence of trials designed to evaluate these interventions within exercise-integrated or function-oriented frameworks. Most studies have prioritized isolated symptomatic outcomes, often without accounting for contextual factors such as physical activity, rehabilitation participation, or functional goals. Addressing these methodological gaps is essential to clarifying whether nutraceuticals have a clinically relevant adjunctive role within exercise-centered care.

Priority 1. Prioritizing patient-centered outcomes beyond pain scores.

Future research on nutraceuticals in KOA should move beyond isolated pain scores as primary endpoints [67]. Although pain remains clinically relevant, outcomes that more directly reflect functional capacity—such as physical performance, habitual activity levels, and participation in exercise-based care—are likely to be more informative in real-world settings [22,23,68]. Measures related to mobility, balance, and falls risk may be particularly relevant in older adults with sarcopenic vulnerability [21].

Importantly, future studies should distinguish between direct symptomatic effects and downstream functional consequences. Without this distinction, modest analgesic effects may be either overstated as independent therapeutic efficacy or underestimated when they support participation in exercise-based care. Aligning outcome selection with functional goals would improve interpretability and facilitate integration with guideline-based KOA management.

Priority 2. Exercise-integrated and context-aware trial designs.

Given the central role of exercise in KOA management, future trials should evaluate nutraceuticals within exercise-integrated frameworks rather than as isolated interventions [11,33,62]. Designs that assess whether nutraceuticals facilitate initiation, adherence, or tolerance of structured exercise programs would more directly test their proposed adjunctive role.

Such approaches may also help explain the heterogeneity observed across existing studies. By accounting for baseline activity levels, pain-related barriers, rehabilitation participation, and sarcopenic vulnerability, trials can better identify patient subgroups in whom adjunctive strategies are most relevant. Context-aware designs would reduce reliance on indirect inference and strengthen causal interpretation without overstating efficacy [69].

Priority 3. Standardization, safety, and implementation-focused research.

Substantial gaps remain in standardization and safety assessment. Future studies should prioritize transparent reporting of product composition, bioavailability, and dosing to enable meaningful comparison across trials [39]. Long-term safety data in older adults with multimorbidity, polypharmacy, or chronic organ dysfunction are particularly needed [64,70].

Equally important is implementation-focused research that examines how nutraceuticals are used in routine clinical practice. Studies addressing patient expectations, clinician decision-making, monitoring strategies, treatment duration, and criteria for discontinuation may be as informative as traditional efficacy trials. Clarifying when nutraceuticals should not be used is essential to preventing overuse and aligning clinical practice with evidence [71].

Beyond the agents reviewed here, certain performance-oriented nutrients warrant exploration as future research candidates rather than current recommendations. For example, L-carnitine, which supports mitochondrial fatty-acid transport and oxidative metabolism, has been associated in healthy adults with improvements in muscle strength, exercise capacity, and fatigue resistance in a systematic review and meta-analysis framed from a rehabilitation perspective [72]. Whether these effects translate to patients with KOA—particularly older adults with reduced mobility, muscle weakness, and sarcopenic vulnerability, in whom oxidative metabolism and fatigue may limit rehabilitation participation—is unknown, as direct KOA-specific evidence is lacking. Dedicated trials with standardized dosing, attention to safety and patient selection, and rehabilitation-oriented functional endpoints would be required before any clinical role could be considered.

Overall, future research should test nutraceuticals not as stand-alone treatments for KOA or sarcopenia, but as optional adjuncts within exercise-centered care. The key unanswered question is whether selected nutraceuticals can meaningfully reduce symptom-related barriers, improve exercise tolerance, and support sustained functional participation in appropriately selected patients.

## 11. Conclusions

Knee osteoarthritis with coexisting sarcopenic vulnerability can be understood as a coupled muscle–joint condition in which pain, physical inactivity, neuromuscular impairment, inflammation, and anabolic resistance interact to drive functional decline. Within this framework, physical function—rather than structural joint change alone—represents the most clinically meaningful outcome.

Exercise-based interventions remain the cornerstone of management, yet sustained participation is frequently limited by pain, reduced tolerance to loading, and diminished physical capacity, particularly in older or vulnerable populations. Addressing these barriers is therefore central to effective care.

Current evidence does not support oral nutraceuticals as disease-modifying therapies in KOA, nor do they consistently provide substantial symptomatic benefit when used in isolation. However, when interpreted within a muscle–joint framework, selected nutraceuticals may be considered as optional, time-limited adjuncts that provide modest symptomatic effects and may reduce barriers to exercise participation in selected patients.

Accordingly, nutraceuticals should not be viewed as stand-alone treatments or as substitutes for structured exercise and rehabilitation. Their potential clinical value lies only in supporting engagement with exercise-based care, and their use should be individualized, goal-directed, and regularly reassessed. Until higher-quality evidence is available, this adjunctive role should be interpreted conservatively within an exercise-centered management strategy. Future studies should use standardized nutraceutical protocols and rehabilitation-oriented outcomes—including exercise adherence, gait performance, muscle strength, physical performance, and sarcopenia-related parameters—to determine whether any adjunctive benefit meaningfully supports exercise-centered KOA care.

## Figures and Tables

**Table 1:** Epidemiology and prognostic association between sarcopenia and knee osteoarthritis.

Study (Year)	Design/Population	Sample Size (*n*)	Definition of Sarcopenia (Framework/Domains/Cutoffs)	Key Findings in KOA/OA Context	Effect Estimates/Heterogeneity Notes
Pegreffi et al. (2023) [6]	Systematic review and meta-analysis (4 cross-sectional studies; KOA vs. controls).	7495 (KOA + controls)	Heterogeneous; mass-only or mass + performance criteria; variably aligned with EWGSOP/EWGSOP2 domains.	Sarcopenia prevalence was higher in KOA than controls, supporting frequent KOA–functional vulnerability overlap. However, study-to-study variation was substantial due to definitional and ascertainment heterogeneity.	Prevalence: 45.2% (KOA) vs. 31.2% (controls); pooled OR 2.07 (95% CI 1.43–3.00) with high heterogeneity (I^2^ = 85%); sensitivity analysis reduced OR to 1.88 after removing an outlier.
Xie et al. (2025) [8]	Systematic review and meta-analysis (27 observational studies; OA overall, predominantly KOA).	21,302 (OA total)	Varied: EWGSOP2, AWGS 2019, FNIH frameworks; mass ± strength ± performance. ^a^ Assessment modalities included DXA, BIA, and SARC-F.	Global prevalence estimates underscore that the ‘signal’ is not a single point prevalence, but a reproducible pattern: OA populations frequently meet criteria for low strength and/or poor performance, which plausibly amplifies disability and rehabilitation barriers.	Pooled prevalence: sarcopenia 16.7% (I^2^ ≈ 99%); sarcopenic obesity 14.0% (I^2^ ≈ 99%). Subgroups: higher prevalence in hospitalized settings (19.2%), age >75 (29.5%), and when using muscle-mass-only criteria (17.0%)—illustrating definitional and setting-driven heterogeneity.
Veronese et al. (2021) [9]	Longitudinal cohort (OAI, USA).	2492	Consensus-domain logic: low SMI (sex-stratified) + chair-stand time >15 s (5 rises) as weakness proxy.	Lower-limb weakness/low mass predicted incident symptomatic KOA and radiographic outcomes, aligning with a muscle–joint vulnerability framework.	Symptomatic KOA: OR 2.29 (95% CI 1.42–3.71); Radiographic KOA: OR 1.44 (95% CI 0.90–2.31).
Gao et al. (2024) [10]	Longitudinal cohort (CHARLS, China; adults ≥60 y).	4980	AWGS 2019; possible sarcopenia vs. confirmed sarcopenia (mass + strength/performance). ^a^	Sarcopenia was associated with increased incident KOA, reinforcing the clinical relevance of functional vulnerability rather than a single prevalence estimate.	Incident KOA: OR 1.91 (95% CI 1.15–3.18); association stronger in females.

This table summarizes key epidemiologic and prognostic associations between sarcopenia and knee osteoarthritis (KOA), drawing from systematic reviews and longitudinal cohort studies. Reported estimates reflect heterogeneity in sarcopenia definitions, diagnostic frameworks, and study populations. Findings should be interpreted as associative rather than causal, highlighting the functional relevance of muscle-related vulnerability in KOA rather than a direct etiologic relationship. ^a^ Representative diagnostic cutoffs by framework: AWGS 2019—handgrip strength < 28 kg (men)/<18 kg (women), gait speed < 1.0 m/s, SPPB ≤ 9, 5-chair-stand ≥ 12 s; FNIH—grip < 26 kg (men)/<16 kg (women), ALM/BMI < 0.789 (men)/<0.512 (women). Abbreviations in the table body: y, years.

**Table 2:** Selected exercise-integrated or function-oriented nutritional adjunct trials relevant to functional engagement in KOA.

Study (Year)	Population	Supplement/Nutritional Adjunct (Dose, Duration)	Exercise Context	Participation-Proxy Outcomes	Symptoms/Function	Safety/Tolerability	Clinical Take-Home
Liao et al. (2021) [22]	Women 60–85 y; KOA (ACR, KL I–III); N = 72; 12 wks + 6-mo follow-up.	Protein powder: ≈31.6 g/day (whey 7.0 g + leucine 2.2 g/serving), twice daily; 12 wks.	Comparator: RET alone. Home-based elastic RET 2×/wk, 12 wks; RPE 13–15.	↑ Walking speed aMD +0.09 m/s; ↑ PA aMD +30.0 MET-h/wk vs. RET alone.	WOMAC global aMD −8.21; pain −1.37; physical difficulty −6.49 vs. RET alone.	No serious AEs; supplement adherence 100%; RET compliance ≈83–84%.	Exercise-integrated adjunct with performance and activity-volume signals; not validated surrogates for adherence.
Wu et al. (2024) [23]	Adults 50–70 y; KOA (KL 1–2) + sarcopenia (AWGS 2019); N = 130 (124 analyzed); 12 wks.	Plant protein/peptide powder: 32 g twice daily, 12 wks (placebo: maltodextrin); includes hyaluronic acid.	Placebo-controlled; no structured exercise (usual activity maintained).	SPPB total Δ +1.03 (95% CI 0.69–1.38; *p* < 0.0001); balance +0.65; gait speed +0.36; chair-stand NS.	WOMAC total Δ −4.19 (95% CI −5.32 to −3.06); pain −1.74; stiffness −0.56; function −1.81 (all *p* < 0.0001).	No serious AEs; AEs unrelated to product.	Supplement-only trial; SPPB signal reflects readiness/capacity, not exercise adherence.
Park et al. (2025) [24]	Adults 40–75 y; KOA (KL I–II); N = 80 (PP: LMCP 29, placebo 31); 180 days.	LMCP: 3000 mg/day (500 mg tabs, 3 tabs twice daily); 180 days.	Placebo-controlled; no exercise co-intervention; follow-ups at days 45/90/135/180.	WOMAC function Δ −4.10 ± 9.64 vs. −0.71 ± 6.47 (*p* = 0.035); PROM-based capacity signal.	WOMAC pain Δ −1.90 ± 4.14 vs. +0.61 ± 3.97 (*p* = 0.006); no JSW or inflammatory marker change.	No AEs; compliance ≈95–97%.	Longer-duration supplement-only RCT; symptom/function improvement may lower exercise barriers; adherence not measured.
Neves Jr et al. (2011) [25]	Postmenopausal women 50–65 y; KOA (ACR); N = 26 (24 analyzed); 12 wks.	Creatine monohydrate: 20 g/day × 7 d (loading), then 5 g/day × 11 wks.	Placebo (dextrose) + identical supervised lower-limb RET 3×/wk, 12 wks.	Timed-stands (30 s): CR 18.1 ± 1.8 vs. placebo 15.2 ± 1.2 (*p* = 0.004).	WOMAC function and stiffness improved (CR only); pain decreased in both groups.	No AEs; creatinine clearance unchanged; exercise adherence ≈79% (CR) vs. ≈82% (placebo).	Performance augmentation during strengthening; timed-stands reflects capacity, not adherence.
Park et al. (2025) [26]	Women 45–65 y; sarcopenia (SMI <5.7 and/or HGS <18 and/or SPPB <9) + cartilage defect (MSC implantation); N = 36; 12 wks.	Beetroot juice nitrate: 70 mL (6.5 mmol NO_3_^−^), 2 h pre-RE session; 24 sessions/12 wks.	Placebo (nitrate-depleted beetroot) + identical RE (NWB 0–6 wks, FWB 6–12 wks).	SPPB interaction time × group *p* = 0.005; placebo declined −1.17 at 12 wks. Knee extension MVIC higher in nitrate group at 6 and 12 wks.	IKDC Δ +8.03 favoring nitrate at 12 wks; WOMAC improved in both groups (NS interaction).	Attendance ≈94.5–94.8% both groups; no major safety signals.	Postoperative context (MSC); capacity-preservation signal (SPPB stability) may support rehab participation; not direct adherence evidence.

This table presents an illustrative rather than exhaustive compilation of selected exercise-integrated or function-oriented nutritional adjunct trials evaluating nutraceutical, protein-based, or performance-oriented supplementation in knee osteoarthritis (KOA). The included studies were selected to represent the range of participation-proxy and capacity-related outcome signals currently available, not to provide a comprehensive inventory of all relevant trials. In this review, participation-proxy outcomes refer to performance, activity-volume, or functional-capacity measures that may reflect readiness or capacity for exercise participation, such as gait speed, Short Physical Performance Battery (SPPB) scores, activity volume expressed as metabolic equivalent task–hours per week (MET-h/week), or performance-based functional tests. These outcomes should not be interpreted as direct measures of exercise adherence or causal effects on exercise behavior. Symptom- and function-related outcomes are included to contextualize these signals. Findings should be interpreted cautiously given the small number of available studies, heterogeneity in study design, supplement formulation, and exercise context. Clinical take-home messages indicate potential adjunctive contexts for exercise-based care, without implying disease modification. For interpretation, commonly cited meaningful-change estimates in older adults are approximately 0.05 m/s (small) and 0.10 m/s (substantial) for gait speed, and approximately 0.5 points (small) and 1.0 point (substantial) for the SPPB [27]. Thresholds for WOMAC and VAS are noted in Table 3. These values contextualize the reported signals, which reflect performance or functional capacity rather than direct measures of exercise adherence. Abbreviations/symbols in the table body: y, years; wks, weeks; mo, months; ↑, increase or improvement relative to comparator.

**Table 3:** Meta-analytic anchors for direct symptomatic effects of nutraceuticals in knee osteoarthritis.

Nutraceutical	Outcome (Meta-Analytic Anchor)	Effect Size vs. Placebo	Clinical Interpretation (KOA MIC/MID/MCII Context)	Confidence/Key Limitations
Glucosamine/Chondroitin	Pain (VAS/WOMAC) SR/MA of RCTs (multiple, e.g., OARSI evidence synthesis [13])	Small and variable (SMD ≈ −0.20 to −0.30 across analyses)	Typically below or near the lower bound of KOA important change thresholds; any average benefit is small relative to MCII/MID ranges and may be formulation-dependent. ^a^	Substantial heterogeneity; strong formulation and study-quality dependence; divergent guideline recommendations
Collagen peptides	Pain SR/MA of RCTs (Lin et al., 2023 [28])	Moderate reduction (SMD −0.58; 95% CI −0.98 to −0.18; I^2^ ≈ 68%)	May approach the lower end of KOA important-change thresholds in selected trials, but average effects remain modest and heterogeneous; interpret as potential barrier-reduction adjunct rather than a primary analgesic. ^a^	Between-study heterogeneity; short follow-up duration; risk of bias and small-study effects
Omega-3 fatty acids	Pain (VAS/WOMAC) SR/MA of RCTs across osteoarthritis populations, including knee OA (Deng et al., 2023 [29])	Small improvement (SMD −0.29; 95% CI −0.47 to −0.11)	Generally below KOA important-change thresholds for pain; effects, when present, are small and unlikely to reach MCII/MID at the group level. ^a^	Heterogeneous EPA/DHA dosing; limited KOA-specific trials; indirect comparisons
Curcumin	Pain and function outcomes, Bayesian network meta-analysis of curcumin therapy for knee osteoarthritis (Zhao et al., 2024 [30])	Short-term improvement versus placebo, including VAS pain MD −1.63 and WOMAC total score MD −18.85 in network meta-analysis.	May provide short-term symptomatic improvement in selected patients, particularly with bioavailability-enhanced formulations. Benefits should be interpreted as symptom-targeted and formulation-dependent, not disease-modifying.	Short follow-up, small-study effects, formulation heterogeneity, and bioavailability dependence limit generalizability.
Boswellia	Pain and function outcomes, systematic review and meta-analysis of Boswellia and Boswellia extract for osteoarthritis (Yu et al., 2020 [31])	Short-term improvements in VAS pain and WOMAC pain, stiffness, and function, with pooled estimates suggesting moderate symptomatic benefit.	May provide short-term symptom relief in selected patients, but the effect is not sufficiently consistent to support broad therapeutic claims or disease-modifying interpretation.	High heterogeneity, variable extract standardization, risk of bias, sponsorship concerns, and limited long-term evidence.
Vitamin D	Pain/function: large randomized trial evidence, not a systematic review/meta-analysis. (MacFarlane et al., 2020 [32])	No consistent symptomatic benefit	Below KOA important-change thresholds; no reproducible advantage over placebo unless correcting deficiency, and functional relevance remains uncertain. ^a^	Baseline deficiency heterogeneity; largely null findings in adequately powered trials

This table summarizes representative meta-analytic effect estimates for commonly used nutraceuticals in knee osteoarthritis, focusing on direct symptomatic outcomes. Effect sizes are presented as anchors to contextualize the typical magnitude of benefit and are interpreted cautiously in relation to reported minimal clinically important difference (MCID) ranges. Key supporting systematic reviews or meta-analyses are cited within the table. Given substantial heterogeneity, formulation dependence, and risk of bias in smaller or short-term studies, these estimates should not be interpreted as evidence of disease modification or definitive clinical efficacy. ^a^ Clinical interpretations marked with this symbol should be read against the following KOA anchor-based thresholds: VAS pain MCII ≈ 10–37 mm (0–100 scale), WOMAC-function MCII ≈ 5–20 points (0–100 scale), varying by baseline severity.

**Table 4:** Clinical positioning of nutraceuticals in knee osteoarthritis with sarcopenic vulnerability.

Nutraceutical Category	Evidence Summary	Functional/Muscle Relevance	Safety/Quality Considerations	Clinical Positioning
Glucosamine/chondroitin	Extensively studied in RCTs, meta-analyses, and guidelines; symptomatic effects are variable, modest, and formulation-dependent.	No direct evidence for muscle mass, strength, or performance benefit in KOA; relevance is indirect via symptom relief.	Generally well tolerated, but product quality varies; interaction caution is warranted, particularly with anticoagulant therapy.	Optional symptom-targeted adjunct; not disease-modifying; trial with predefined reassessment interval.
Collagen peptides	Meta-analyses suggest modest pain and function improvement; effects vary by formulation, dose, and duration.	KOA-specific muscle evidence is limited; exercise-related functional relevance is largely extrapolated from non-KOA populations.	Short-term tolerability is generally favorable; peptide source, composition, dose, and bioavailability vary substantially.	Functional-support adjunct for symptomatic patients; should not imply cartilage regeneration or structural restoration.
Omega-3 fatty acids	Evidence for KOA symptom relief is small or inconsistent; anti-inflammatory rationale is biologically plausible but not clearly disease-specific.	Muscle-related signals derive from non-KOA populations; KOA-specific strength or performance benefit is not established.	EPA/DHA composition, dose, background diet, and comparator oils vary; caution in patients with atrial fibrillation risk or anticoagulant/antiplatelet use.	Selected adjunct when broader metabolic or inflammatory context supports use; not a primary KOA-specific therapy.
Curcumin/Boswellia	Short-term RCTs and meta-analyses suggest possible pain relief, especially with bioavailability-enhanced or standardized formulations.	Functional gains may parallel pain reduction; objective performance or muscle-specific KOA evidence remains sparse.	Formulation, bioavailability, extract standardization, and dose vary; gastrointestinal, hepatic, and drug-interaction concerns should be considered.	Short-term symptom adjunct, particularly when conventional analgesic options are constrained; periodic reassessment required.
Vitamin D	No consistent KOA-specific symptomatic benefit in unselected patients; clinical relevance depends largely on baseline deficiency.	May support bone–muscle health when deficient; not established as a KOA-specific exercise-enabling intervention.	Avoid excessive dosing; monitor in patients at risk of hypercalcemia, renal disease, or relevant medication interactions.	Correct deficiency when present; should not be presented as a KOA treatment in vitamin-D-replete patients.
Protein-rich/amino-acid–based supplementation	Exercise-integrated or function-oriented trials suggest potential relevance in older adults with KOA and/or sarcopenic features.	Most relevant to participation proxies (gait speed, SPPB, activity volume, chair-stand), especially when combined with exercise.	Consider renal function, baseline nutritional status, total protein intake, dose, and tolerability.	Potential exercise-supportive adjunct in older patients with sarcopenic vulnerability, particularly when nutritional adequacy is uncertain.
Creatine/nitrate, L-carnitine, and other performance-oriented candidates	Evidence remains limited and often comes from small, selected, exercise-integrated, postoperative, or non-generalizable contexts. L-carnitine shows performance- and fatigue-related benefits in healthy adults but no KOA-specific evidence.	Some performance signals exist, but evidence is insufficient for routine KOA use or direct anti-sarcopenic claims.	Patient selection, renal/cardiovascular considerations, and long-term safety require caution.	Future research candidates; not recommended as routine KOA nutraceuticals without clearer evidence and selection criteria.

Abbreviations and note. KOA, knee osteoarthritis; RCT, randomized controlled trial; SPPB, Short Physical Performance Battery; EPA, eicosapentaenoic acid; DHA, docosahexaenoic acid. This table summarizes clinical positioning rather than formal treatment recommendations. Nutraceuticals should not be interpreted as disease-modifying therapies, direct anti-sarcopenic interventions, or substitutes for structured exercise and rehabilitation.

**Table 5:** Reported formulation, dose range, duration, and outcome signals for selected nutraceuticals in knee osteoarthritis.

Nutraceutical	Formulation Studied	Reported Dose Range	Reported Timing and Duration	Observed Outcome Signals (Key Caveat)
Glucosamine	Pharmaceutical-grade crystalline glucosamine sulfate; OTC salts and HCl preparations heterogeneous	1500 mg/day (single dose or 500 mg ×3)	Oral, daily; ~6 mo–3 y	Small, formulation-dependent pain/function signal; no consistent structural effect (caveat: warfarin-interaction signal)
Chondroitin	Pharmaceutical-grade chondroitin sulfate; OTC variable	800–1200 mg/day	Oral, daily; ~6 mo–2 y; often combined with glucosamine	Small-to-moderate pain signal in some analyses; glucosamine/chondroitin combination non-inferior to celecoxib in one 6-month trial [34] (caveat: heterogeneity, formulation dependence, sponsorship)
Collagen peptides	Hydrolyzed collagen or low-molecular-weight collagen peptides (LMCP); source/hydrolysis vary	Wide range (LMCP 3000 mg/day [24]; collagen hydrolysate ~10 g/day)	Oral, daily; ~3–6 mo	Modest, heterogeneous pain/function signal; no established structural modification (caveat: small, product-specific trials with frequent industry sponsorship)
Omega-3 fatty acids	Marine EPA + DHA (fish oil); EPA/DHA ratio and concentration vary	~1–4 g/day combined EPA + DHA	Oral, daily with food; ~3–24 mo	Small/inconsistent KOA symptomatic signal despite systemic anti-inflammatory plausibility; muscle effects mainly from non-KOA populations (caveat: EPA/DHA heterogeneity; higher-dose atrial-fibrillation signal)
Curcumin	Bioavailability-enhanced formulations (phospholipid complexes, nanoparticles, or piperine-containing); unmodified preparations vary	Highly formulation-dependent (~180–1500 mg/day)	Oral, daily; ~4–12 wk	Short-term pain/function signal, particularly with enhanced formulations (caveat: rare hepatotoxicity reports)
Boswellia serrata	Standardized extract (e.g., AKBA-enriched); standardization varies	~100–250 mg/day standardized extract (or ~1000 mg crude)	Oral, daily; ~4–12 wk	Short-term symptomatic improvement in some trials/meta-analyses (caveat: high heterogeneity, sponsorship concerns)
Vitamin D	Cholecalciferol (D3)	Deficiency correction guided by serum 25(OH)D	Oral, daily or weekly until repletion	No consistent KOA-specific symptomatic signal in unselected patients; relevant mainly for deficiency correction and general musculoskeletal health (caveat: avoid excess/hypercalcemia)

Values represent dose ranges and intervention durations reported in selected cited studies and should not be interpreted as formal dosing recommendations or clinical prescribing guidance. Substantial heterogeneity in formulation, dose, bioavailability, comparator, and treatment duration limits cross-trial comparability. Observed effects are generally modest, formulation-dependent, and not disease-modifying. Nutraceuticals are positioned in this review as optional, time-limited adjuncts within exercise-centered care. Protein- and performance-oriented adjuncts, including protein/leucine, creatine, and nitrate, are summarized within their exercise-integrated trial contexts in Table 2. OTC, over-the-counter; HCl, hydrochloride; LMCP, low-molecular-weight collagen peptides; EPA, eicosapentaenoic acid; DHA, docosahexaenoic acid; AKBA, acetyl-11-keto-β-boswellic acid; 25(OH)D, 25-hydroxyvitamin D. Abbreviations in the table body: mo, months; y, years; wk, week.

## Data Availability

No new data were created or analyzed in this study. Data sharing is not applicable to this article.

## Supplementary Materials

The following supporting information can be downloaded at: https://www.mdpi.com/article/10.3390/nu18121871/s1, Figure S1: Flow diagram of study identification, screening, eligibility assessment, and inclusion.
