# Supplementary material for: Reframing Nutraceuticals in Knee Osteoarthritis with Sarcopenia: A Muscle–Joint-Centered Narrative Review
Source: Nutrients. 2026 Jun 10;18(12):1871. doi: 10.3390/nu18121871 (PMC13304766; doi:10.3390/nu18121871)
Supplement: Supplementary file 1 [file nutrients-18-01871-s001.zip › nutrients-4360711-supplementary.pdf]

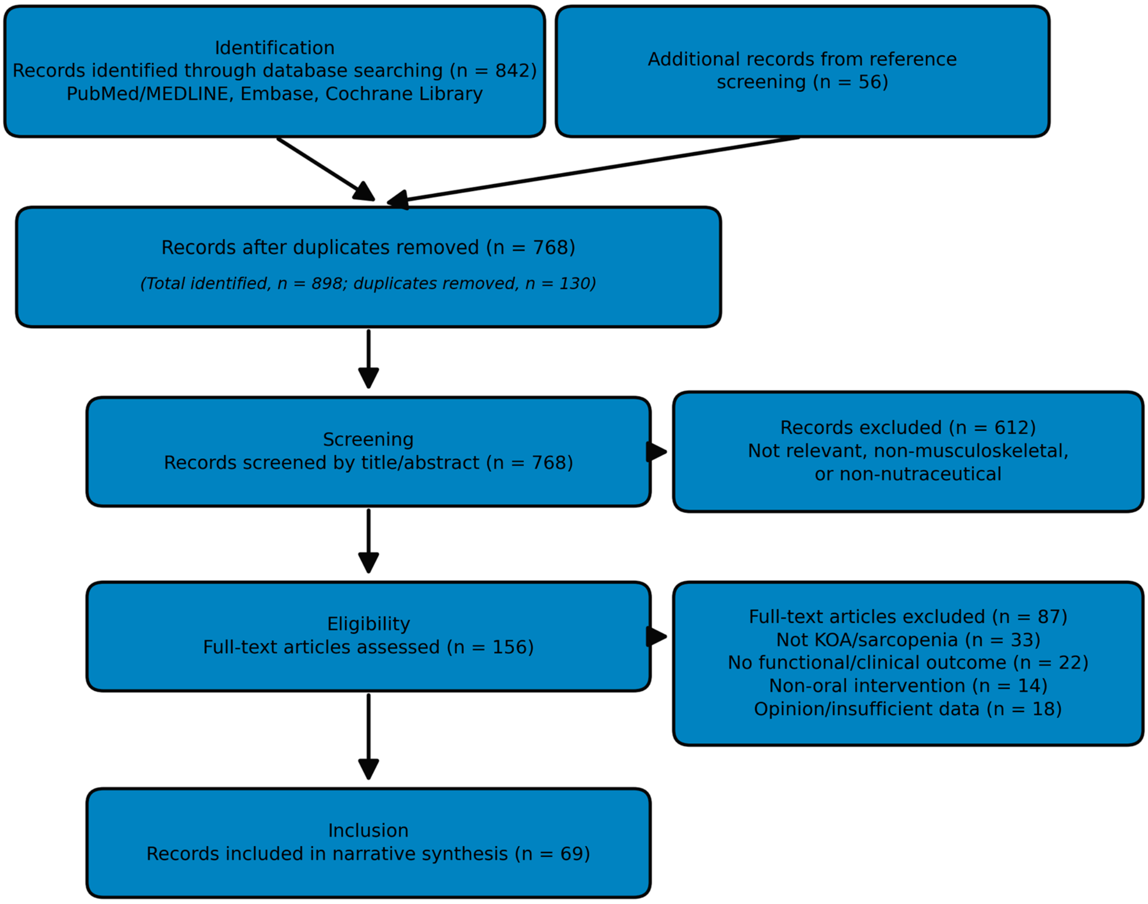

Figure S1. Flow diagram of study identification, screening, eligibility assessment, and inclusion. Flow of records through the structured narrative review. Records were identified through database searching (PubMed/MEDLINE, Embase, and the Cochrane Library) and reference screening, de-duplicated, screened by title and abstract by two independent reviewers, and assessed in full text against the eligibility criteria, yielding 69 sources that informed the narrative synthesis. The diagram documents the identification and selection process; the synthesis itself was qualitative and narrative, without quantitative pooling, formal per-study risk-of-bias assessment, or formal certainty-of-evidence grading.
